# *ZmDST44* Gene Is a Positive Regulator in Plant Drought Stress Tolerance

**DOI:** 10.3390/biology13080552

**Published:** 2024-07-23

**Authors:** Wenbo Chai, Hongtao Li, Hanyuan Xu, Qing Zhu, Shufen Li, Chao Yuan, Wei Ji, Jun Wang, Lei Sheng

**Affiliations:** 1 Lianyungang Academy of Agricultural Sciences, Lianyungang 222006, China; 13215605136@163.com (W.C.); hongtaoli1987@163.com (H.L.); xuhanyuan1988@126.com (H.X.);; 2 Anhui Academy of Agricultural Sciences, Hefei 230036, China

**Keywords:** maize, drought tolerance, *ZmmiR139*, *ZmDST44*

## Abstract

**Simple Summary:**

Improving drought tolerance in plants is crucial for increasing crop yields, especially in water-limited environments. We studied a maize gene called ZmDST44, which is regulated by a specific miRNA named *ZmmiR139*. Our experiments showed that *ZmmiR139* controls ZmDST44 by cutting its mRNA. When we overexpressed ZmDST44 in *Arabidopsis*, rice, and maize, the plants became more resistant to drought. These transgenic plants had lower levels of stress indicators, higher levels of protective compounds, and increased expression of genes that help them cope with drought. Our findings suggest that ZmDST44 is an important gene for enhancing drought tolerance in plants, offering potential benefits for developing drought-resistant crops through genetic engineering.

**Abstract:**

Improving drought tolerance in plants is essential for increasing crop yields under water-limited conditions. In this study, we investigated the functional role of the maize gene ZmDST44, which is targeted by the miRNA *ZmmiR139*. Our results indicate that *ZmmiR139* regulates ZmDST44 by cleaving its mRNA, as confirmed by inverse expression patterns and 5′-RACE analysis. Overexpression of ZmDST44 in Arabidopsis, rice, and maize resulted in significant enhancements in drought tolerance. Transgenic plants exhibited reduced malondialdehyde (MDA) levels, increased proline accumulation, and upregulation of drought-responsive genes compared to wild-type plants. Transgenic *Arabidopsis* and rice showed improved drought resistance and higher post-drought recovery rates, and transgenic maize displayed lower sensitivity to drought stress. These findings suggest that ZmDST44 acts as a positive regulator of drought tolerance across different plant species and holds promise for developing drought-resistant crops through genetic engineering.

## 1. Introduction

Drought is one of the most important environmental factors that can affect a plant because it can inhibit photosynthesis and decrease plant growth and productivity. Drought stress impacts many aspects of maize growth. For example, drought stress during the seeding and flowering stages has been estimated to cause a 13% loss in grain yield [1]. Water stress reduces the production of new leaves and promotes senescence and abscission [2]. Stomatal control of water loss is an early plant response to drought, but this reduces the influx of carbon dioxide. Lower internal CO_2_ concentrations during drought cause reductions in photosynthesis levels by affecting the acceptor site of the ribulose-1, 5-bisphosphate carboxylase/oxygenase (RuBisCo) enzyme and by directly inhibiting photosynthetic enzymes such as RuBisCo or ATP synthase [3]. 

Ribulose-1,5-bisphosphate carboxylase/oxygenase is the most abundant protein on earth [4]. It is an enzyme responsible for the fixation of atmospheric carbon dioxide and regulates photosynthesis and photorespiration in C3 plants. Improving the catalytic properties of this enzyme increases crop productivity [5]. Plant RuBisCo is composed of eight catalytic large subunits (LSU) and eight small subunits encoded by a nuclear multigene family; the large subunit is encoded by the chloroplast rbcL gene [6]. In senescent tissues, the activity of RuBisCo is decreased [7,8]. RbcL can be divided into an N-terminal domain (residues 1–50) and a C-terminal domain (residues 151–475), and its two active sites are located at the interface of the rbcL subunits within the L2 dimers [9]. When the plastid rbcL gene in the plastid genome is deleted, transgenic plants exhibit a severe RuBisCo deficiency [10]. 

MicroRNAs (miRNAs) are 21-nt single-stranded RNA molecules that are known to act as major post-transcriptional regulators of various developmental pathways and stress responses and that act by negatively regulating their target genes through transcript cleavage or translational inhibition miRNAs [11,12,13,14,15]. They are transcribed from long stem-loop precursor RNAs in plants and processed by Dicer-like 1 (DCL1) before the mature miRNA molecules are incorporated into RNA-induced silencing complexes (RISC) [16,17,18]. In plants, many miRNA families are conserved in monocots and dicots, and these miRNA genes are usually highly expressed and produced by several genetic loci [19]. For example, the miR169 family has been identified in more than 40 species and plays an important role in plant development and responses to abiotic stressors [20,21,22]. Furthermore, a number of target miRNAs genes are important transcription factors and play crucial roles in plant growth and development [23,24]. Dicotyledonous plants have 21 conservative miRNA families. Of the 95 target genes involved, 65 are important transcription factors. Therefore, miRNAs are indispensable regulatory factors in plants [25].

Maize is one of the most important crops in the world and is also an essential raw material for the food, fuel, and fodder industries [26]. Drought stress, one of the major causes of yield reduction in maize, is an important area of investigation for breeders and researchers [27]. High-throughput sequencing approaches to studying genes related to drought resistance have helped us understand the maize drought resistance mechanism and create new drought-resistant maize varieties [28]. In this study, we identified the novel miRNA PC-5p-139812 (ZmmiRNA139) and its target gene *ZmDST44*. *ZmDST44* encodes diphosphoribulose carboxylase in chloroplasts, and its KEGG biochemical metabolic pathways include glyoxylate and dicarboxylate metabolism, carbon fixation, and carbon metabolism. The expression of miRNA and *ZmDST44* exhibited an inverse correlation [29]. We found that the ectopic expression of *ZmDST44* in *Arabidopsis*, rice, and maize plants was critical for drought stress tolerance in these plants. Together, our results suggest that *ZmDST44* has a conserved positive regulator function in plant responses to drought stress.

## 2. Materials and Methods

### 2.1. Plant Material and Growth Conditions

Seeds from two inbred maize lines, Hz4 (drought-tolerant) and 3189 (drought-sensitive), were germinated in a greenhouse and grown under standard conditions until the seedlings had developed three leaves. The seeds for the maize inbred lines and *Oryza sativa* L. ssp. Japonica seeds were obtained from the Biophysics Key Laboratory of Anhui Agricultural University, and the *Arabidopsis thaliana* seeds were Col-0 wild type. Seedlings were grown in a growth chamber under a 14 h day/10 h night photoperiod at 28–30 °C. For the drought stress experiments, seedlings were grown until they had developed three leaves. For the ABA treatment experiments, seedlings were grown on MS medium supplemented with 0, 3, 4, or 5 µM ABA and then incubated at 4 °C for 5 days and then at 28 °C. For the PEG treatment experiment, seedings were grown on MS medium with 200 mM PEG6000 for 15 days at 28 °C.

### 2.2. 5′ RACE Identification of the miRNA Target Cleavage Site 

A GenRacer Kit (Invitrogen, Waltham, MA, USA) was used to perform 5′-RACE. Total RNA was isolated from plants with three leaves, and the GeneRacer RNA Oligo was ligated to the 5′ end of the mRNA. Oligo dT primers were used to synthesize first-strand cDNA, and then the initial PCR was carried out using the 5′ primer and gene-specific primers. A 1-µL sample was collected after the initial PCR and used as template for the nested PCR using nested primers. The amplicons were then cloned into pEASYT1 and sequenced. The gene-specific primers are listed in Table S1.

### 2.3. Construction of Overexpression Vectors 

After cloning *ZmDST44* from the Hz4 and 3189 maize inbred lines, we subcloned the purified PCR products into the pEASY-T1 Cloning Vector and transformed the resulting recombinant plasmid into *E. coli* strain DH5α. Subsequently, the recombinant plasmid was extracted, and the entire plasmid was sequenced. We then cloned the *ZmDST44* fragment into pCAMBIA1301 under the control of the 35S promoter. Finally, the combined *ZmDST44* gene was transferred into plants using Agrobacterium-mediated plant transformation processes [30].

### 2.4. Transformation of Rice and Maize

The lowland japonica rice cultivar ‘Zhonghua 11’ was used for transformation. After surface sterilization of the seeds with NaClO, callus was induced at 26 °C induction medium. Agrobacterium (GV3101) cells were collected, suspended in liquid medium, and the concentration of Agrobacterium cells was adjusted to OD600 = 0.3–0.5. Acetosyringone (final concentration 100 mM) was added to the Agrobacterium suspension for the co-cultivation and transformation of rice [31].

The maize embryos and Agrobacterium (EHA105) were used as materials for transformation. After infection, the callus tissues were transferred to differentiation culture medium until the plants were grown. 

### 2.5. Germination and Root Length Assays

The *Arabidopsis* seeds were sterilized with 12% NaClO for 15 min and then washed with water several times. The seeds were sowed on MS agar plates with ABA, which were incubated at 4 °C for 3 days, and then kept at 22 °C. The germination rate of the seeds was calculated after 10 days.

For the root growth length and lateral root number assays, the WT and *ZmDST44* plants were sowed on the MS plates. After 4 days of growth, the *Arabidopsis* plants were transferred to new plates containing 30 µM, 40 µM, or 50 µM ABA. The lengths of the seedlings were measured after 10 days of upright growth in the treatment medium.

### 2.6. Drought Stress Treatments on Transgenic Plants

For drought stress tolerance evaluation, WT and transgenic seedlings were transplanted into soil and grown in a greenhouse under standard conditions. After the plants reached the five-leaf stage, they were not watered again for 15 days. After 15 days, watering was resumed, and then the plants were examined for the development of new green leaves. Any plants that developed new green leaves were considered to have survived the drought. 

### 2.7. RNA Extraction, Quantitative RT-PCR

Total RNA was isolated from *Arabidopsis* frozen tissue samples using TRizol reagent (TaKaRa, Osaka, Japan). We used quantitative RT-PCR (qRT-PCR) to quantify the expression levels of PsaL and other stress genes in wild-type and transgenic *Arabidopsis* (over-expressed *ZmDST44*). The AtActin was used as control gene. Fold changes in expression were estimated using the 2^−ΔΔCT^ method [32]. Expression data are reported as mean ± SE for three independent replications. The qRT-PCR primers for the PsaL and stress genes are listed in Table S2.

### 2.8. Determination of Malondialdehyde (MDA) Content

Samples were collected from the same parts of the third leaves before and after drought-stress treatment. Each sample was analyzed with three biological replicates. Briefly, a small amount of quartz sand and 5 mL of 0.05 M phosphate buffer (pH 7.5) (V1) were added to a mortar. Next, 0.5 g of leaf material (W) was added to the mortar and ground using a pestle. The homogenate was centrifuged at 6000 rpm for 15 min. An aliquot (2 mL) of the supernatant (V2) was mixed with 3 mL of 0.5% (*w*/*v*) TBA in 5% TCA. The solution was incubated at 100 °C for 15 min and then rapidly cooled. After centrifugation at 6000 rpm for 10 min, the absorbance at 532 and 600 nm (A532 and A600) was determined, using water as the control. The MDA content was calculated using the extinction coefficient (155 mM^−1^ cm^−1^) with the following formula:

MDA (nmol L^−1^) = (A532 − A600) × V1 × V/1.55 × 10^−1^ × W × V2

### 2.9. Relative Electrolyte Leakage Estimation

Samples were collected from the same parts of the third leaves before and after drought-stress treatment. All leaf samples were incubated in 20 mL ddH_2_O in tubes with evacuation for 5 h at 25 °C until the samples had sunk to the bottom of the tube. After shaking for 5 h at 25 °C, electrolytic leakage (K1) was measured using a DDS-11A conductivity meter (Leizi, Shanghai, China). The sample tubes were boiled for 10 min at 100 °C in a water bath and then rapidly cooled. The electrolyte leakage of the boiled samples (K2) was measured at 25 °C. The electrolytic leakage of sterile ddH_2_O (K0) was measured as the control. The relative electrolyte leakage (REL) was estimated using the following formula: REL (%) = (K1 − K0)/(K2 − K0) × 100. Each sample was analyzed using three biological replicates.

### 2.10. Determination of Proline Content

Proline was extracted and quantified following the method of Bates et al. [33]. Leaf segments were homogenized in 3% sulfosalicylic acid and the homogenate was centrifuged at 3000× *g* for 20 min. The supernatant was treated with acetic acid and acid ninhydrin, boiled for 1 h, and then the absorbance at 520 nm was determined. The proline content was expressed as mmol g^−1^ fresh weight (FW).

### 2.11. DAB Staining

After drought-stress treatment, a 2 cm segment of the third leaf was excised at a distance of 5 cm from the leaf tip. The leaf sample was placed in a 15 mL centrifuge tube covered with aluminum foil, to which either 10 mL diaminobenzidine (DAB) staining solution or 10 mM NaHPO4 (as a control) was added. The centrifuge tubes were placed horizontally on a shaker at 80 rpm for 4–5 h. The DAB solution was replaced with a bleaching solution (ethanol:acetic acid:glycerol (*v*/*v*/*v*), 1:1:1), then the solution was incubated at 90–95 °C in a water bath for 15 min and then at room temperature for 30 min. Brown precipitates formed by the reaction of DAB with hydrogen peroxide were observed and recorded when placed on the culture medium.

### 2.12. Statistical Analysis

Experimental data for the morphological and physiological characteristics of the treated plants were analyzed using GraphPad Prism software 7.0. Data are herein presented as the mean ± standard deviation of three replicates. The significance of any differences between the WT and transgenic plants was assessed using Student’s *t*-test.

## 3. Results

### 3.1. ZmDST44 mRNA Is Cleaved by ZmmiR139

**Figure 1:**
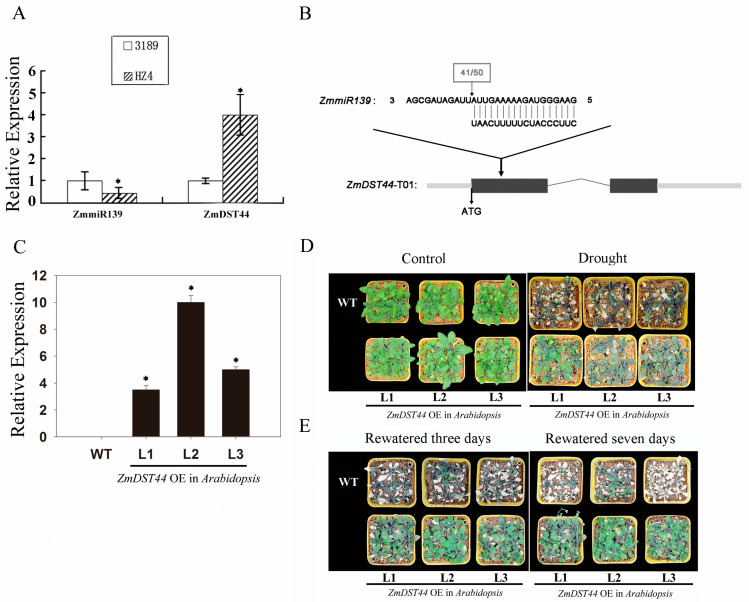
Phenotypic changes in wild-type and transgenic *Arabidopsis* under drought treatment and after rewatering. (**A**) Relationship between ZmmiRNA139 and its target gene, *ZmDST44*. (**B**) *ZmDST44* mRNA cleavage is directed by *ZmmiR139*; arrows above the target mRNA indicate the 5′ ends of cleavage products. (**C**) Detection of *ZmDST44* mRNA in *ZmDST44* transgenic Arabidopsis. (**D**) Drought treatment; (**E**) rewatering for 3 and 7 days. WT: wild type plants; L1–L4: different lines of transgenic Arabidopsis. * *p* < 0.05, (Student’s *t*-test).

To elucidate the functions of *ZmmiR139* in *ZmDST44*, we used RT-PCR to compare the levels of *ZmDST44* transcripts in 3189 (drought-senstive) and HZ4 (drought-tolerant). In 3189, *ZmDST44* transcript levels were reduced, but *ZmmiR139* was upregulated; whereas, transcript levels were high in HZ4 *ZmDST44*, but *ZmmiR139* was downregulated (Figure 1A). These observations suggested that *ZmmiR139* might be involved in the cleavage of *ZmDST44*. These results also indicated that the expression of ZmmiRNA139 and its target gene *ZmDST44* are inversely related. To determine whether *ZmmiR139* can cleave *ZmDST44* mRNA in vivo, 5′-RACE was used to verify the interaction between *ZmmiR139* and *ZmDST44*. We found that most cleavage sites mapped to the target regions (Figure 1). Therefore, we concluded that *ZmDST44* was the target gene for *ZmmiR139*.

### 3.2. Overexpression of ZmDST44 Confers Drought Tolerance in Arabidopsis

**Figure 2:**
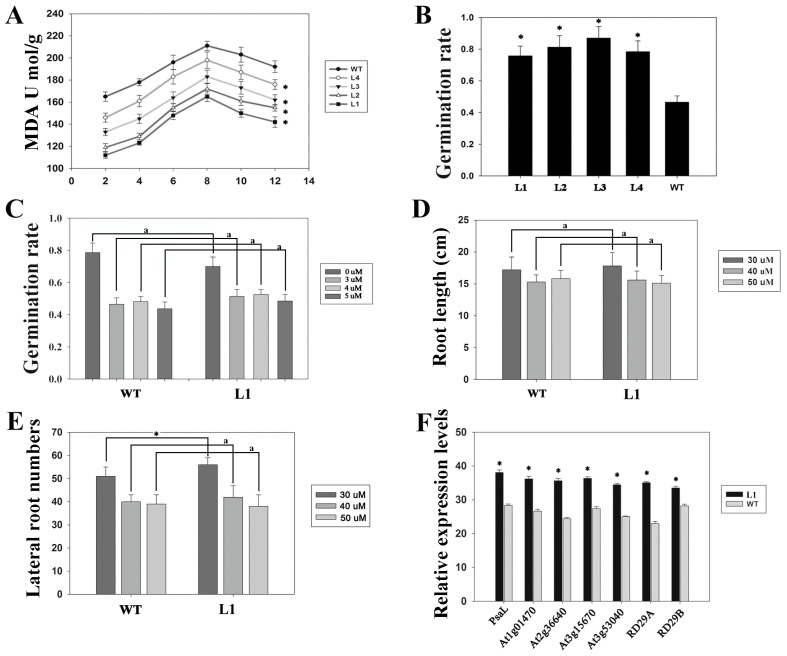
Improved tolerance to drought stress in *ZmDST44* transgenic *Arabidopsis*. (**A**) The MDA content of wild-type plants and different transgenic *Arabidopsis* under drought treatment; (**B**) the germination rates of wild-type and different transgenic seeds after treatment with PEG 6000; (**C**) the germination rate of wild-type and transgenic seeds after treatment with different concentrations of ABA; (**D**) the root length of wild-type *Arabidopsis* and transgenic *Arabidopsis* after treatment with different concentrations of ABA; (**E**) the lateral root number of wild-type plants an *Arabidopsis* after treatment with different concentrations of ABA. WT: wild-type plants; L1–L4: different lines of transgenic Arabidopsis. (**F**) The relative expression quantity of PsaL and several other genes induced by stress in transgenic *Arabidopsis* and wild-type Arabidopsis. Values represent the mean ± SD of triplicates. AtActin was used as a control gene. * *p* < 0.05, (Student’s *t*-test). “a” means there was no difference between the two sets of data.

The T2 generation seedlings of wild-type and transgenic *Arabidopsis* were subjected to drought-stress treatment. As shown in Figure 1D, at the beginning of the treatment, there were no notable differences between transgenic and wild-type plants. However, the transgenic plants showed a more drought-resistant phenotype after the 9-day drought treatment. After 3 days of rewatering, most of the transgenic plants recovered. However, only a few of the wild-type plants survived for 7 days (Figure 1E). Moreover, the levels of malondialdehyde (MDA) in the different transgenic plants subjected to the drought treatment were significantly lower than those in wild-type *Arabidopsis* plants (Figure 2A). We also investigated the germination rates in *Arabidopsis* of wild-type and transgenic seeds after treating them with PEG 6000, and we found that the germination rate of the transgenic seeds was significantly higher than that of the wild-type seeds (Figure 2B). We then investigated how the seeds germinated on MS culture media containing 30 µM, 40 µM, or 50 µM ABA solutions. As shown in Figure 2D, there was no significant difference in the root length of the wild-type and transgenic plants after exposure to any of the ABA concentrations. These results suggested that *ZmDST44* might be involved in drought resistance in an ABA-independent way.

### 3.3. Drought-Related Genes Are Highly Expressed in Plants Overexpressing of ZmDST44

To investigate the roles *ZmDST44* may play in drought tolerance pathways, we used RT-PCR to measure the transcript levels of several genes involved in the dehydration response. The expression of PsaL, which can increase drought tolerance, was strongly induced in transgenic plants [34]. We found that the expression levels of PsaL and other stress genes in transgenic *Arabidopsis* were significantly higher than those in wild-type Arabidopsis. This indicated that the overexpression of *ZmDST44* could significantly increase the expression levels of PsaL and other stress genes (Figure 2F).

### 3.4. ZmDST44 Transgenic Rice Exhibits an Enhanced Tolerance to Drought

**Figure 3:**
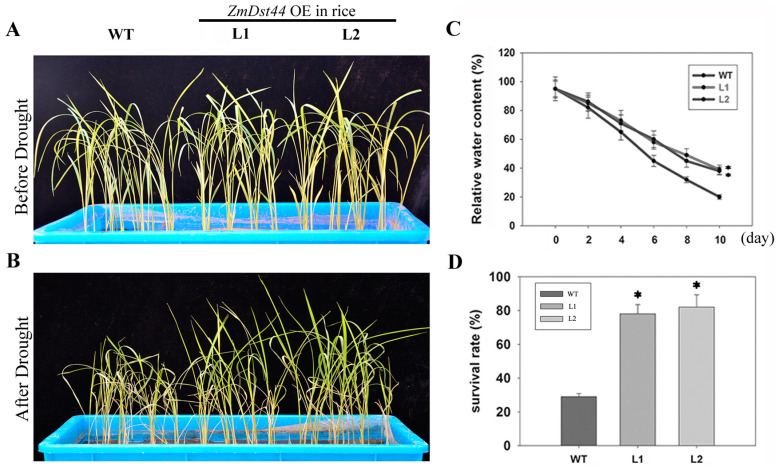
Phenotype changes in wild-type plants and transgenic plants before and after drought treatment. The relative water content and survival rate of wild-type plants and transgenic plants under drought treatment is also shown. (**A**) Rice plants under normal conditions; (**B**) rice plants after drought treatment; (**C**) relative water content of leaves; (**D**) survival rates of wild-type plants and transgenic plants. WT: wild-type plants; L1, L2: lines of transgenic rice. Values represent the mean ± SD of triplicates. * *p* < 0.05, (Student’s *t*-test).

To investigate the function of *ZmDST44* in monocotyledons, we generated transgenic rice that harbored full-length *ZmDST44*. Under well-watered conditions, there were no significant differences between the wild-type rice plants and those that overexpressed *ZmDST44*. However, the transgenic rice was remarkably resistant to dehydration under drought conditions (Figure 3A,B). The relative water contents (RWC) of both plants were measured, and the RWC of transgenic rice was found to be higher than that of wild-type rice after the drought treatment. This suggests that the overexpression of *ZmDST44* can improve the water retention of plants under drought stress conditions. The transgenic rice was observed to have a high survival rate after re-watering (Figure 3). Taken together, these results suggested that the function of *ZmDST44* in drought resistance is conserved in rice.

### 3.5. Overexpression of ZmDST44 Enhanced Maize Tolerance to Drought Stress

**Figure 4:**
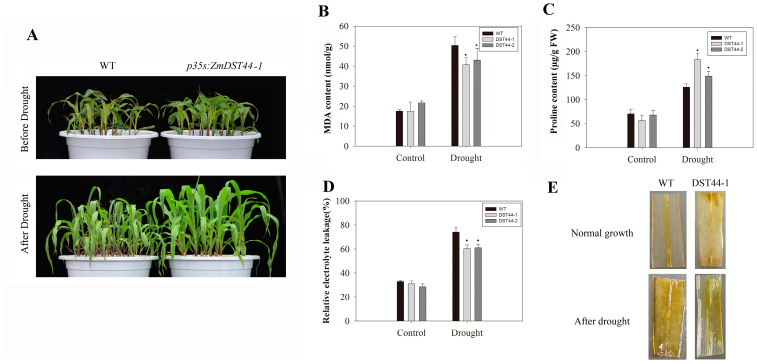
Phenotype analysis of wild-type and transgenic maize before and after drought stress. (**A**) Maize plants under normal condition; maize plants under drought treatment; (**B**) MDA content; (**C**) relative electrolyte leakage; (**D**) proline content of wild-type plants. WT: wild type, p35s:*ZmDST44*: transgenic plants that over expressed *ZmDST44*. (**E**) DAB staining. Values represent the mean ± SD of triplicates, * *p* < 0.05, (Student’s *t*-test).

We then investigated the function of *ZmDST44* in maize and generated over 10 transgenic maize plants containing Pro35S:*ZmDST44*. Both the *ZmDST44* transgenic and wild-type plants showed similar phenotypes under normal conditions (Figure 4A). However, the *ZmDST44* transgenic plants were less sensitive than the wild-type plants when subjected to drought stress (Figure 4A). The MDA levels in the transgenic plants were lower than those in the wild-type plants, and the proline content of the transgenic plants was higher than that of the wild-type plants after drought treatment; at the same time, the DAB staining of the transgenic plants was lighter than WT (Figure 4B–E). These results indicated that *ZmDST44* might act as a positive regulator in drought-stress tolerance and is a potential factor for consideration in maize breeding.

## 4. Discussion

Inducing excessive gene expression in transgenic organisms is a common method used for studying gene function, whether the genes are exogenous or endogenous. In this study, we cloned *ZmDST44* from the 3189 maize inbred line and used transformation to create transgenic *Arabidopsis*, *rice*, and maize plants that overexpressed *ZmDST44*.

Plants have their own regulatory networks that have developed through long-term evolution processes and allow them to adapt to different kinds of environmental stresses. Active oxygen species levels in plants increase under some abiotic stressors, and this can result in lipid peroxidation and peroxide stress [35,36]. Malondialdehyde content is commonly used to evaluate the extent of plant lipid peroxidation [37]. Our study results revealed that the MDA levels in different transgenic *Arabidopsis* during the drought treatment were significantly lower than those in wild-type plants (Figure 2A), indicating that the overexpression of *ZmDST44* could reduce the levels of membrane lipid peroxides in transgenic *Arabidopsis*. Moreover, the overexpression of *ZmDST44* might improve the ability of plants to retain water under drought stress (Figure 2E). These results showed that overexpression of *ZmDST44* could significantly improve drought tolerance in transgenic *Arabidopsis.*

**Figure 5:**
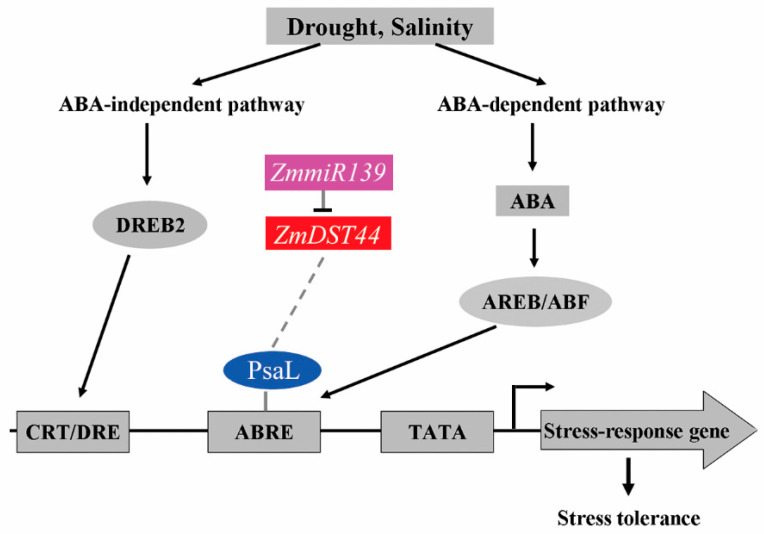
Regulation network response to drought stress in which *ZmmiR139* and *ZmDST44* may be involved.

A previous study reported that AtFKBP16-1, which encodes immunophilin in chloroplasts, could also mediate photosynthesis stress by regulating the stability of the PsaL protein, a subunit of the photosystem I (PS I) complex [34]. The PsaL gene codes for important subunits in plant photosystem I, and the PsaL promoter has many cis-regulatory elements [38]. These include ABA response components (ABRE). The accumulation of PsaL protein in transgenic *Arabidopsis* that overexpressed AtFKBP16-1 was significantly higher than that in wild-type *Arabidopsis*, and the drought resistance of the transgenic plants was also increased. Based on these results, we performed quantitative RT-PCR to quantify the expression of PsaL and other stress genes that overexpressed *ZmDST44* in transgenic *Arabidopsis* and wild-type Arabidopsis. We found that the expression levels of PsaL and other stress genes in transgenic *Arabidopsis* were significantly higher than those in wild-type *Arabidopsis*, which indicated that the overexpression of *ZmDST44* could significantly improve the expression levels of PsaL and other stress genes (Figure 2F). Therefore, we can predict that *ZmmiR139* and *ZmDST44* may be involved in the regulation network responsible for responding to drought stress. As shown in Figure 5, *ZmmiR139* could affect the accumulation of the PsaL protein by regulating the expression level of *ZmDST44*. When the expression level of *ZmDST44* significantly increased, this led to the accumulation of high levels of PsaL protein. Then, the PsaL proteins would combine with ABA response components (ABRE) to activate the expression of drought-stress-induced genes and enhance the drought resistance of plants (Figure 5).

In transgenic rice, the survival rate is higher than in wild lines. Phenotypic data showed that the growth of transgenic rice plants after drought treatment was better than that of the wild-type plants. This shows that overexpression of *ZmDST44* could significantly improve drought tolerance in transgenic *Rice.*

To see the stress effect of drought, we measured the MDA content, proline content and ROS. Many ROS are produced when plants are stimulated by drought stress, which triggers metabolic disorder. ROS-mediated oxidative stress can cause biofilm peroxidation, and any malondialdehyde (MDA) produced can damage biofilms and lead to high amounts of electrolyte permeation. Under drought stress, the proline content of the plant increased, and this accumulation enhanced plant tolerance. In transgenic maize, the MDA level was lower than that in the control maize, and the proline content of the transgenic maize was higher after drought treatment; at the same time, the DAB staining of the transgenic plants was lighter. These results indicated that *ZmDST44* might act as a positive regulator in drought stress tolerance in maize.

All results showed these transgenic plants were more resistant to dehydration, suggesting that *ZmDST44* might play a more general role in dehydration resistance. They also suggested that the involvement of *ZmDST44* in the drought stress regulatory network might be conserved among different species. These experiments related to genetic modification were all conducted in the laboratory, which is significantly different from practical applications in production. Our findings not only provide better resources for stress resistance improvement by crop genetic engineering, but also lay the foundation for analyzing the molecular mechanism of maize drought resistance.

## 5. Conclusions

In this study, we characterized the role of the maize gene *ZmDST44*, which is targeted by the miRNA *ZmmiR139*, in enhancing drought tolerance in plants. Our results demonstrate that *ZmmiR139* regulates *ZmDST44* by cleaving its mRNA, as evidenced by the inverse relationship between *ZmmiR139* and *ZmDST44* expression levels and confirmed by 5′-RACE analysis.

Overexpression of *ZmDST44* in *Arabidopsis* conferred significant drought tolerance, as evidenced by reduced malondialdehyde (MDA) levels and enhanced expression of drought-responsive genes. Transgenic rice exhibited improved drought resistance and higher survival rates compared to wild-type plants. Furthermore, transgenic maize plants displayed lower sensitivity to drought stress, MDA level was lower than that in the control maize, and the proline content of the transgenic maize was higher after drought treatment; at the same time, the DAB staining of the transgenic plants was lighter. All results indicate a conserved role of *ZmDST44* in monocot and dicot plants, which can increase tolerance to drought.

These findings suggest that *ZmDST44* is a critical positive regulator of drought stress tolerance, potentially acting in an ABA-independent manner. The conserved function of *ZmDST44* across different plant species highlights its potential utility in developing drought-resistant crops through genetic engineering. Future research could further explore the underlying mechanisms of *ZmDST44*-mediated drought tolerance and its application in crop improvement programs.

## Data Availability

We declare that all of the gene transfer work in our experiment is not expected to cause any harm. All raw data will be available if needed.

## Supplementary Materials

The following supporting information can be downloaded at: https://www.mdpi.com/article/10.3390/biology13080552/s1, Table S1: The specific primers for 5′-RACE. Table S2: qRT-PCR primers for PsaL and some genes induced by adversity stress.
