# Supplementary material for: ZmDST44 Gene Is a Positive Regulator in Plant Drought Stress Tolerance
Source: Biology (Basel). 2024 Jul 23;13(8):552. doi: 10.3390/biology13080552 (PMC11351533; doi:10.3390/biology13080552)
Supplement: Supplementary file 1 [file biology-13-00552-s001.zip › biology-3082057-supplementary.pdf]

Table S1 The specific primers for 5’-RACE

Primer	Sequence
GeneRacer 5′ Primer	5-CGACTGGAGCACGAGGACACTGA-3
GeneRacer 5′ Nested Primer	5-GGACACTGACATGGACTGAAGGAGTA-3
Reverse 448344 primer	5- ATAATCCTTAACACCAGC -3
Reverse 448344 nested primer	5- CTACCAAAGTTTCGCTACCTATGGTAT -3

Table S2 QRT-PCR primers for PsaL and some genes induced by adversity stress.

**Gene**	**Forward primer**	**Reverse primer**
At4g12800	ACAAGACAACATTCCAAGTAG	GAGGAGAGGGTTGACGGCGGT
At1g01470	TGGTGTGGACTGGGACATTGA	TAGCAAACCCAACTTATTACA
At2g36640	TGCTCGGAAAGATAAGGGAAA	AAACAAGAATACAAACATAGA
At3g15670	GAAGACAAAGAGCATTATCCA	AGTATTTGTTGTTGTAGTAGA
At3g53040	GAGAATCGTCGTGGGAGAGAA	TAGTTTTATTACAAGTCAAGA
RD29A	AACACACACCAGCAGCACCCA	GTAATCGGAAGACACGACAGG
RD29B	AGGAAGGAAAAGGTGGAGAAG	GGAATCCGAAAACCCCATAGT
